# Asymmetrical predation intensity produces divergent antipredator behaviours in primary and secondary prey

**DOI:** 10.1111/1365-2656.14166

**Published:** 2024-08-28

**Authors:** Corbin C. Kuntze, M. Zachariah Peery, Jonathan N. Pauli

**Affiliations:** ^1^ Department of Forest and Wildlife Ecology University of Wisconsin Madison Wisconsin USA

**Keywords:** antipredator behaviour, asymmetrical predation, habitat domain, hunting mode, optimal foraging, risk management

## Abstract

1. It is widely recognized that predators can influence prey through both direct consumption and by inducing costly antipredator behaviours, the latter of which can produce nonconsumptive effects that cascade through trophic systems. Yet, determining how particular prey manage risk in natural settings remains challenging as empirical studies disproportionately focus on single predator–prey dyads.
2. Here, we contrast foraging strategies within the context of a primary and secondary prey to explore how antipredator behaviours emerge as a product of predation intensity as well as the setting in which an encounter takes place.
3. We studied the effects of spotted owls (*Strix occidentalis*) on two species experiencing asymmetrical risk: dusky‐footed woodrats (*Neotoma fuscipes*; primary prey) and deer mice (*Peromyscus* spp.; alternative prey). Woodrats are most abundant within young forests, but predominantly captured by owls foraging within mature forests; in contrast, deer mice occur in high densities across forest types and seral stages and are consumed at lower per‐capita rates overall. We deployed experimental foraging patches within areas of high and low spotted owl activity, created artificial risky and safe refuge treatments, and monitored behaviour throughout the entirety of prey foraging bouts.
4. Woodrats were more vigilant and foraged less within mature forests and at riskier patches, although the effect of refuge treatment was contingent upon forest type. In contrast, deer mice only demonstrated consistent behavioural responses to riskier refuge treatments; forest type had little effect on perceived risk or the relative importance of refuge treatment. Thus, habitat can interact with predator activity to structure antipredator responses differently for primary versus secondary prey.
5. Our findings show that asymmetrical predation can modulate both the magnitude of perceived risk and the strategies used to manage it, thus highlighting an important and understudied contingency in risk effects research. Evaluating the direct and indirect effects of predation through the paradigm of primary and secondary prey may improve our understanding of how nonconsumptive effects can extend to population‐ and community‐level responses.

## Graphical abstract

These findings demonstrate that among a diverse prey guild within a spatially heterogeneous environment, asymmetrical predation between a primary and secondary prey species can modulate both the magnitude of perceived risk and the strategies used to manage it, highlighting an important and understudied contingency in risk effects research.

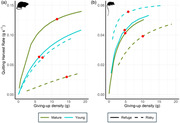

## 1 INTRODUCTION

Predator–prey interactions can be likened to an adaptive foraging game where each participant strives to outwit the other (Brown et al., 2001; Kotler, 2016). In this context, the success of the predator is determined by the ability to encounter and subdue their prey, while the success of the prey is determined by the ability to avoid and escape their predators (Brown et al., 2001; Wolf & Mangel, 2007). Prey species have honed their strategies to navigate this high‐stakes arena through morphological, behavioural, and physiological defences (Brown & Kotler, 2004; Schmitz & Trussell, 2016). While these adaptations increase the likelihood of immediate survival of prey, they often involve nonconsumptive effects (NCEs), or trade‐offs with other consequences that influence fitness (Preisser et al., 2005; Wirsing et al., 2021)—the most fundamental being the balance between food and safety (Brown & Kotler, 2004). The implications of NCEs are not confined to the predator–prey dyad; the propagation of these risk effects is now widely recognized for their ability to drive higher order interactions that can rival or even exceed consumptive effects in terms of their impact on ecosystem structure and function (Donadio & Buskirk, 2016; Wirsing et al., 2021).

Antipredator behaviours emerge from complex spatiotemporal dynamics as the outcome of both background and immediate levels of perceived risk (Lima & Bednekoff, 1999; Moll et al., 2017). For prey, the immediate risk of predation is typically driven by direct cues from predators that inform the likelihood of encounter or capture (Creel, 2011; Lima & Dill, 1990). This acute risk may increase with proximity or activity levels of predators (Kohl et al., 2019), while the presence of conspecifics among prey may attenuate risk through cooperative vigilance, defence, or dilution effects (Alexander, 1974; Carthey & Banks, 2015). In contrast, prey often lack the ability to directly detect the presence or densities of their predators (Lima & Dill, 1990), and rely on indirect cues to evaluate chronic background risk (Gaynor et al., 2019). This gives rise to the ‘landscape of fear’, which can generate spatiotemporal variability in trait responses and NCEs within prey communities (Gaynor et al., 2019; Laundré et al., 2014). Prey, then, manage these complex risk dynamics by adopting both proactive and reactive strategies (Lima & Bednekoff, 1999; Wirsing et al., 2021). This can be seen in foraging behaviours, as prey balance the trade‐offs between food and safety with behavioural titrations (Brown & Kotler, 2004; Kotler & Blaustein, 1995), including patch selection, time allocation, and apprehension (Brown & Kotler, 2004; Kotler et al., 2010). For example, some animals prioritize foraging within patches with greater food availability or with lower risk (Brown & Kotler, 2004), while others may practice vigilance to increase safety at the cost of foraging efficiency (Kotler et al., 2010). Understanding these decisions can help us assess how foragers behave optimally by balancing the marginal costs and benefits of foraging (Kotler & Blaustein, 1995).

Investigations of risk effects have largely centred around spatiotemporal properties of the environment that influence the likelihood of predator‐induced mortality; however, a growing body of literature suggests that properties of the organisms involved may also contribute to the nature and magnitude of antipredator behaviour (Schmitz & Trussell, 2016; Wirsing et al., 2021). Within diverse prey communities, perceived risk can also vary with the presence or absence of other prey and relationships with the predators themselves (Holt & Lawton, 1994; Wilson et al., 2022). For example, by specializing in one or several preferred species from a pool of potential prey, dietary preferences among predators can drive differences in predation intensity between these ‘primary’ and alternative ‘secondary’ prey species (Holt & Lawton, 1994). Within the context of multiprey systems, this interplay between primary and secondary prey species introduces more complexity that further modulates risk beyond morphology, state dependencies, or spatiotemporal variation within the landscape of fear (Gaynor et al., 2019). While the ecological consequences of shared natural enemies have been explored in the context of consumptive effects and apparent competition (Holt & Lawton, 1994), empirical studies on risk effects disproportionately focus on single predator–prey pairings and provide an incomplete picture of contingencies in NCEs (Sheriff et al., 2020; Wirsing et al., 2021). A few notable studies have explored these dynamics in multipredator or multiprey systems (e.g., Dellinger et al., 2019; Dröge et al., 2017; Kachel et al., 2023). For example, Dellinger et al. (2019) found that two different species of deer (*Odocoileus* spp.) exhibited divergent strategies of space use to terrain suiting their respective running gaits when exposed to grey wolf (*Canis lupus*) predation, demonstrating how prey escape mode can drive spatial variability in the effectiveness of antipredator strategies among sympatric species (Wirsing et al., 2010). However, still missing are empirical studies within multiprey systems that (A) consider how dietary specialization by a predator can modify risk between a primary and secondary prey species (sensu Holt & Lawton, 1994), and (B) examine how differential predation intensity can drive risk management through foraging behaviours.

To explore how properties of predator, prey, and their environment interact to determine perceived risk from a relatively specialized predator, we studied the effects of spotted owls (*Strix occidentalis*) on two prey species that experience differing predation intensities—dusky‐footed woodrats (*Neotoma fuscipes*—primary prey) and *Peromyscus* spp. (alternative prey). Woodrats are the largest‐bodied and most energetically profitable prey for spotted owls, and when present, typically comprise the majority of spotted owl diet in both quantity and total biomass (Kuntze et al., 2023; Zulla et al., 2022). Accordingly, the consumption of woodrats has been linked to benefits in fitness, occupancy, and space use for spotted owls (Hobart et al., 2019; Kuntze et al., 2023). In contrast, smaller bodied secondary prey such as *Peromyscus* spp., voles (*Microtus* spp.), moles (*Scapanus* spp.), and gophers (*Thomomys* spp.) are less important to spotted owls in terms of both number and biomass, although *Peromyscus* spp. are the most frequently consumed among these secondary prey species. *Peromyscus* spp. are also up to 30× more abundant than woodrats (Fraik et al., 2023; Kelt et al., 2017), suggesting that per‐capita predation rates on woodrats are far greater than any alternative prey. Further, while *Peromyscus* spp. are habitat generalists and occur in high densities across different forest types and seral stages (Kelt et al., 2017), woodrats and spotted owls exhibit divergent habitat preferences that should, in theory, limit encounter rates between these two species. Specifically, spotted owls typically nest, roost, and forage in larger patches of mature forest (Jones et al., 2018), while woodrats are associated with—and reach greatest densities within—younger, brushier forests (Kuntze et al., 2023; Sakai & Noon, 1993), traditionally viewed as less suitable habitat for foraging owls.

**FIGURE 1:**
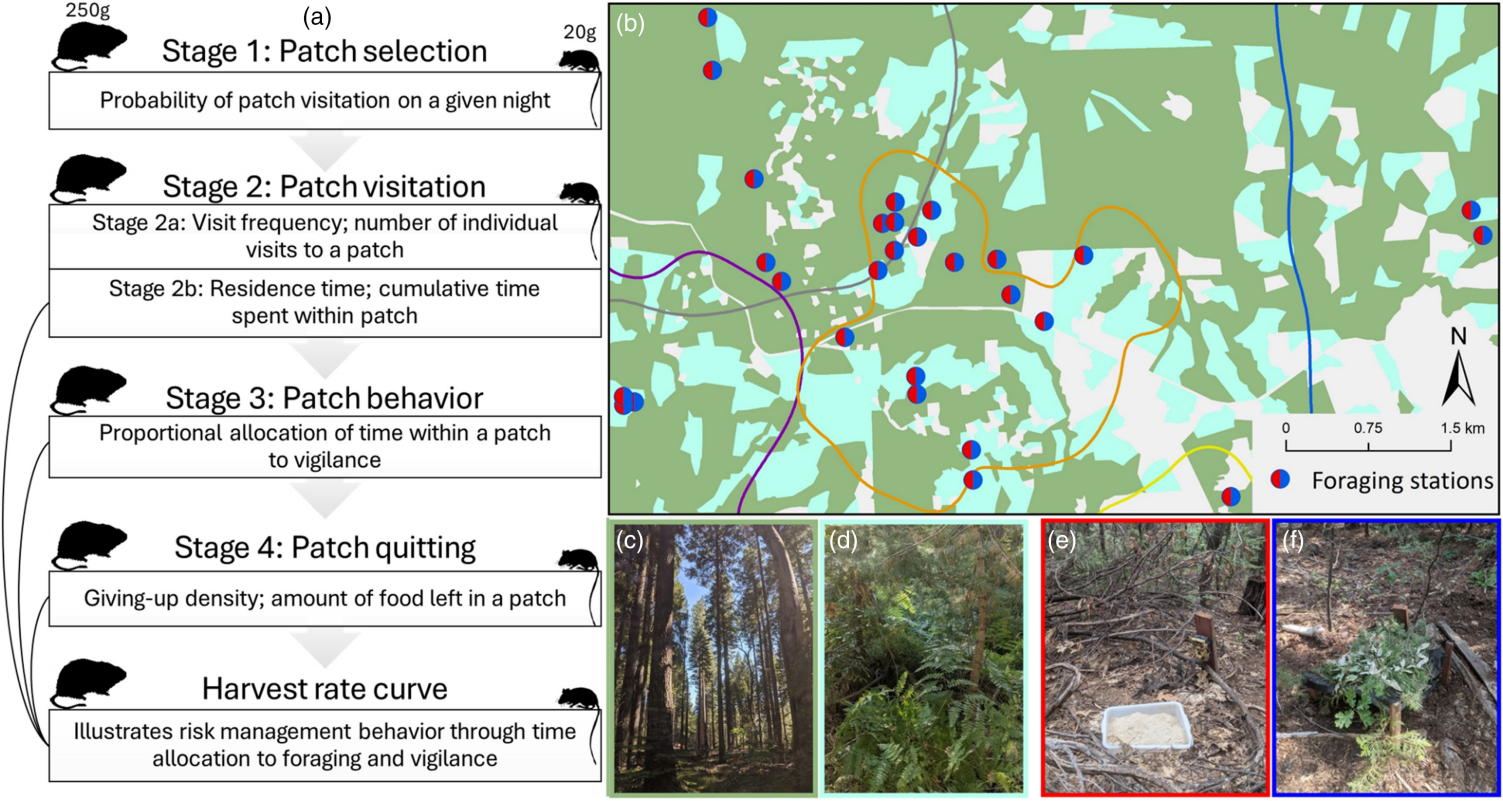
Overview of the study design for exploring antipredator behaviour among dusky‐footed woodrats (*Neotoma fuscipes*) and deer mice (*Peromyscus* spp.) within the central Sierra Nevada, CA, USA. The conceptual diagram (a) outlines the response variables quantified at each stage of the foraging process and the harvest rate curve which synthesizes behaviours and values from stages 2–4. Also shown are the average mass and specific foraging stages explored for both woodrats (left) and deer mice (right). The locations of foraging stations (b) are shown within 95% kernel home ranges (coloured outlines) of GPS‐tagged spotted owls (*Strix occidentalis*) relative to mature (c; green) and young (d; turquoise) forests. Each foraging station consisted of two individual patches with either a risky (e) or refuge treatment (f).

Using spotted owls, woodrats, and *Peromyscus* spp. in a spatially heterogeneous environment featuring variable predation intensity, we contrasted foraging behaviours within the hierarchy of a primary and secondary prey species. Here, we tested whether woodrats and *Peromyscus* spp. perceive risk from spotted owl predation differently and explore the strategies used to manage risk. We hypothesized that the nature and strength of risk experienced by prey—and in turn, the amount of antipredator investment—is governed by both the dietary preferences of the predator and the setting in which an encounter takes place. We predicted that perceived risk would follow spatial patterns of actual risk; that is, we expected that antipredator behaviour would increase in mature versus young forests. We predicted that both prey species would manage risk through behavioural changes at distinct stages of the foraging process (Figure 1a) but expected that the actual strategies employed would differ between the two. Specifically, we predicted that woodrats would exhibit a greater overall antipredator response and that they would manage risk predominantly through decisions on where and how to forage (i.e., patch selection, apprehension, and patch quitting), while *Peromyscus* spp. would reduce the frequency and duration of patch visitations. To test these predictions, we deployed experimental foraging patches within areas of high‐ and low‐spotted owl hunting activity, created artificial risky and safe microhabitat conditions and then monitored woodrat and *Peromyscus* spp. behaviour throughout the entirety of foraging bouts. By systematically exploring these predictions through each stage of the foraging process, we provide further insights into risk perception and contingencies in NCEs by exploring the properties of both predator and prey, as well as the environment in which these interactions occur.

## 2 MATERIALS AND METHODS

### 2.1 Study system

We conducted this study within and adjacent to the Eldorado Demography Study Area, a long‐term spotted owl monitoring site that encompasses ~355 km^2^ of the Eldorado National Forest on the western slope of the central Sierra Nevada, CA, USA. *Peromyscus* spp. here could be one of two species, *P. maniculatus* or *P. boylii*; we hereafter refer to them as deer mice. This study area has been described in detail elsewhere (Kuntze et al., 2023; Zulla et al., 2022), but briefly, land use practices have created a landscape with distinct spatial variation in forest structure and configuration. Here, relatively homogeneous stands of mature mixed‐conifer trees are interspersed with a mosaic of stand types and seral stages—including patches of brushy young forest. The differential use of this landscape among spotted owls, woodrats, and deer mice likely affects these primary and secondary prey differently, further modulating perceived risk and concomitant response.

### 2.2 Field methods

In summer 2021, we conducted foraging experiments within five known and presently occupied adult spotted owl home ranges (Figure 1b). This study was performed with approval by the Institutional Animal Care and Use Committee of the University of Wisconsin, Madison (IACUC #A006173‐A01), and followed guidelines from the American Society of Mammalogists (Sikes & the Animal Care and Use Committee of the American Society of Mammalogists, 2016). Our field methods, particularly site selection, were focused on woodrats due to their status as the primary prey species of spotted owls and their patchy distribution (Kuntze et al., 2023; Sakai & Noon, 1993). Deer mice occurred ubiquitously and at high densities throughout the area. Therefore, our study was designed with the assumption that effective selection of woodrat habitat would effectively include deer mice as well.

We captured and uniquely marked woodrats and then selected a subsample of these individuals (*n* = 73) to fit with VHF collars equipped with onboard mortality sensors (details in Appendix S1). Within 2 days of collar deployment, we tracked woodrats to their middens. We assigned each midden to one of two forest types based on canopy cover and size of dominant trees in the area: mature forest (>40% canopy cover and trees >12 inches diameter at breast height [DBH]; Figure 1c) or young forest (>40% canopy cover and saplings or trees <12 inches DBH; Figure 1d). Additional forest types were grouped into the ‘other’ category and not considered in this study, although open area (<40% canopy cover) accounted for the majority of the ‘other’ category. From these, we selected sites (*n* = 31) within young (*n* = 14) and mature (*n* = 17) forests to capture a gradient of local habitat features, minimize proximity to water or human activity, and allow adequate separation between stations (>150 m) to ensure independence (Figure 1b). Woodrats demonstrate midden fidelity and small home ranges (Sakai & Noon, 1997), which we confirmed by GPS collaring individuals during pilot work in summer 2019. Further, we confirmed that none of the individuals in our study moved during the course of the study by VHF monitoring outside of the midden every 1–2 days.

At each site, we placed a foraging station consisting of two foraging patches—spaced 5 m apart—10 m from a midden that was presently occupied by a collared woodrat. To construct each patch, we first placed a plastic tray in a shallow hole so that the top lip was approximately level with the ground. One patch was randomly chosen as the risky treatment (Figure 1e), while the other was chosen as the safe refuge treatment (Figure 1f). For the risky patch, we cleared all brush <2 m tall from within 1 m of the tray. For the refuge patch, we created a 0.5 × 0.5 m wooden structure over the tray covered with black netting that stood ~40 cm from the ground and mimicked natural cover by thoroughly covering this structure with cut brush. By creating vertical but not horizontal cover at refuge patches, we provided protection from the risk of avian predation but not from mammalian or other cursorial predators (Embar et al., 2014; Makin et al., 2017). We filled each tray with 5 L of sifted sand and 20 g of shelled, halved peanuts; this specific food resource and ratio was determined after field testing to ensure consumption and avoid saturation or depletion following one night of foraging. The peanuts were thoroughly mixed within the matrix to ensure the food resource was buried by sand at differing depths; to standardize any effect of odour on patch selection, one peanut was placed at the surface of the matrix for every tray.

We allowed for 2 days of acclimation in which trays were placed and refilled, but data were not collected. After this, we collected data for a minimum of four consecutive days. Risk perception—and consequent response—may be sensitive to temporally variable conditions (e.g., Prugh & Golden, 2014); we controlled for this by only collecting data on nights with high lunar illumination (>0.5), minimal cloud cover, and no measurable precipitation. Data were collected daily; every morning, sand from each tray was sieved, and recovered food items were dried, cleaned of debris, and then weighed to the nearest centigram (0.01 g). At dusk, a new matrix of 20 g peanuts and 5 L sand was placed within each tray.

Each tray was monitored with infrared, motion‐sensing video cameras (Campark IP66), which recorded at continuous 30‐s intervals until movement ceased. In addition to providing data on visitation and behaviour within the patch, cameras helped address a number of shortcomings associated with traditional approaches to quantifying foraging behaviour (Bedoya‐Perez et al., 2013). Specifically, they provided a reliable method to confirm patch visitation by target individuals and account for the effect of nontarget foragers. In addition, for woodrats, by conducting experiments exclusively on collared individuals we could identify multiple conspecific foragers, even when visitation was not concurrent.

There are few potential alternative predators in this system beyond spotted owls. We only detected grey fox (*Urocyon cinereoargenteus*) at our foraging stations, and at infrequent rates (1.1% of total tray‐nights). We removed these data from all analyses and also removed any nights where the camera malfunctioned or the tray was disturbed by other nontarget species. We inspected all footage to see whether a patch was visited on a given night by either species, then quantified behaviour at foraging patches using program BORIS (Friard & Gamba, 2016). For each experimental night, we recorded the number of patch visits and the cumulative visit duration—hereafter patch residence time (PRT)—by target woodrats and deer mice. We also recorded whether a patch was visited, the number of patch visits, and PRT for all nontarget (i.e., uncollared) woodrats. For target woodrats, we further quantified patch behaviour by recording the duration of time spent foraging (e.g., digging, exploring the patch, and handling food), vigilant (i.e., paused foraging with head up and clear inspection of surroundings), and other (e.g., grooming or interacting with other organisms). We did not quantify these behaviours for deer mice because the mean visit duration was short ($\bar{x}$ = 14.7 s ± 0.76 SE) compared with woodrats, which had average patch visitations that were approximately four times as long ($\bar{x}$ = 57.8 s ± 3.97 SE; Figure S1). The brief visitations and sporadic activity of deer mice prevented us from reliably classifying behaviour into ‘foraging’ or ‘vigilance’.

Following data collection, we conducted vegetation sampling at every foraging station. We centred circular plots with a radius of 12.5 m at the midpoint between paired trays, within which we recorded canopy cover using a densitometer and understory with a visual estimation of the proportion of ground obscured by brush <2 m height. Finally, for each tree within our plot, we recorded species, DBH, and condition (live, dead) and then estimated the total basal area.

### 2.3 Analytical methods

We explored several response variables for the foraging process, separated into four principal stages (Figure 1a). These included patch visitation probability (Stage 1), number of patch visits and PRT (Stage 2), foraging behaviour (Stage 3), and patch quitting (Stage 4). For each, we evaluated the effect of forest type (young, mature) and patch treatment (refuge, risky) targeted as part of our study design, plus a number of predictor variables related to local habitat features and inter‐ and intraspecific foragers, including a unique site ID for each foraging station as a random effect in all models (Table S1). For woodrats, we also included covariates for among‐individual differences in age, sex, and body condition. To quantify body condition, we used residuals from a regression of body mass against hind foot length (Schulte‐Hostedde et al., 2005) for all woodrats captured during the field season (*n* = 195). We also evaluated several a priori interactions derived from our study design and prior literature highlighting habitat features important to woodrats (Fraik et al., 2023; Sakai & Noon, 1993, 1997).

For patch visitation probability (Stage 1), we created a binary response variable for whether each foraging patch was visited (1) or not (0) during a given experimental night. We constructed mixed‐effects logistic regression models and included predictor variables for habitat, intrinsic, and binary variables for visitation by nontarget species and/or nontarget foragers (Table S1).

For each response variable in stages 2–4, we used two statistical approaches. First, we explored only the a priori relationships between refuge treatment and forest type that were targeted as part of our study design. Here we compared the differences between risky and safe refuge treatments within mature and young forest. Next, we constructed separate linear mixed‐effects models for all response variables except the number of nightly patch visits, for which we used a negative binomial linear mixed model. Within each model set, we included all predictor variables from stage I, as well as covariates for the frequency and duration of inter‐ and intraspecific foraging by woodrats and deer mice (Table S1).

To quantify foraging behaviour, we divided the duration of time spent exercising vigilance by PRT to get the percentage of time that a woodrat spent vigilant during a given experimental night as our response variable, then square root‐transformed these values to normalize the data. To avoid outliers from nights with brief visitation, we only included nights in which PRT was >90 s. As stated previously, we did not describe foraging behaviour for deer mice.

We quantified patch quitting using the giving‐up density approach (GUD; Brown, 1988)—testing a response variable for the amount of food (g) remaining in a patch after an experimental night in which the target individual visited (i.e., nights without visitation were not included in the dataset). To address the effect of foraging by nontarget species on the value of remaining food, we calculated values for the total and proportional PRT by the target and nontarget species relative to all foragers for each night. If the nontarget species foraged for >90 s or made up >10% of total PRT, then we removed this night from our dataset. Therefore, we also excluded any predictor variables for deer mice in woodrat models of GUD, or any variables for woodrats in deer mice models.

We conducted analyses in R (R Core Team, 2023) using package lme4 and lmerTest for model construction and package sjPlot for visualization of effect sizes and relationships. We standardized all continuous variables. To address multicollinearity, we excluded highly correlated covariates (|*r*| >0.6) from the same model (Dormann et al., 2013). For each response variable, covariates were tested univariately, then combinations of competitive covariates were determined from a priori hypotheses in a final model set. We ranked models using Akaike Information Criterion adjusted for small sample sizes (AICc). Any model that outperformed the null and was within 2 ΔAICc of the top model (Morin et al., 2020) was deemed competitive. In the results, we report effect sizes and coefficient estimates from the top‐performing models. We followed the same methodology for both species, save for excluding stage III and several predictor variables for deer mice; specifically, as we could not differentiate between individuals from camera data, we omitted variables for conspecific foraging, sex, age, and body condition from model sets.

### 2.4 Behavioural analysis: Harvest rate curve

With values for GUD and PRT at each patch, we estimated quitting harvest rates (QHR) and plotted harvest rate curves sensu Kotler and Brown (1990) and Kotler et al. (2010). We used PRT and GUD data for values of the total time spent in a patch (*t*), plus initial (*N*_0_) versus remaining (*N*_f_) resource density. With video data from foraging individuals, we directly observed and estimated average handling times (*h*; time [g s^−1^] required by the forager to process and consume food) for both woodrats (6 s g^−1^) and deer mice (16 s g^−1^). We subset the data into groups relative to each of our categorical predictor variables (e.g., forest type, treatment, and the interaction of forest type and treatment) and produced values of attack rate (*a*; food encounter rate), mean GUD, and characteristic QHR for comparison.

Finally, we plotted harvest rate curves for each species and forest type–refuge treatment combination using the appropriate estimate of the attack rate from each group and the overall value of *h*. The harvest curve characterizes patch depletion and summarizes risk management strategies; different slopes correspond to distinct levels of apprehension, while the location of the average GUD on the curve represents the characteristic QHR and corresponds to time allocation (Kotler & Brown, 1990). For example, steeper slopes indicate faster harvesting of equal food densities, meaning foragers prioritize harvesting over predator detection, corresponding to less apprehensive animals, whereas lower GUDs on the harvest rate curve represent greater time allocation to feeding.

## 3 RESULTS

We collected 356 experimental tray‐nights of data. After removing 82 nights that were disturbed, occurred during cloudy and rainy periods, or had camera‐related issues, we ultimately evaluated 274 experimental tray‐nights across 28 foraging stations (i.e., 56 unique trays). These were relatively evenly split among forest type (mature = 146; young = 128) and treatment (refuge = 138; risky = 136).

### 3.1 Stage 1: Patch selection

Woodrats visited foraging patches during 202 tray‐nights. Patch visitation probability was greater among females (*β*_male_ = −3.11 [95% CI = −5.83, −0.40]), on nights where a tray was visited by another woodrat (*β*_nontarget_ = 1.07 [0.10, 2.05]), and greater at safe versus risky patches in mature forests (*β*_refuge_ = 1.87 [0.70, 3.03]), but lower at safe versus risky patches in young forests (*β*_young: refuge_ = −2.82 [−4.66, −0.98]; Figure S2; Table S2). Deer mice visited foraging patches during 156 tray‐nights. The top model was the intercept model while no other covariates were informative (Table S3), suggesting that patch visitation probability was unaffected by any tested predictor variables.

### 3.2 Stage 2: Patch visitation

**FIGURE 2:**
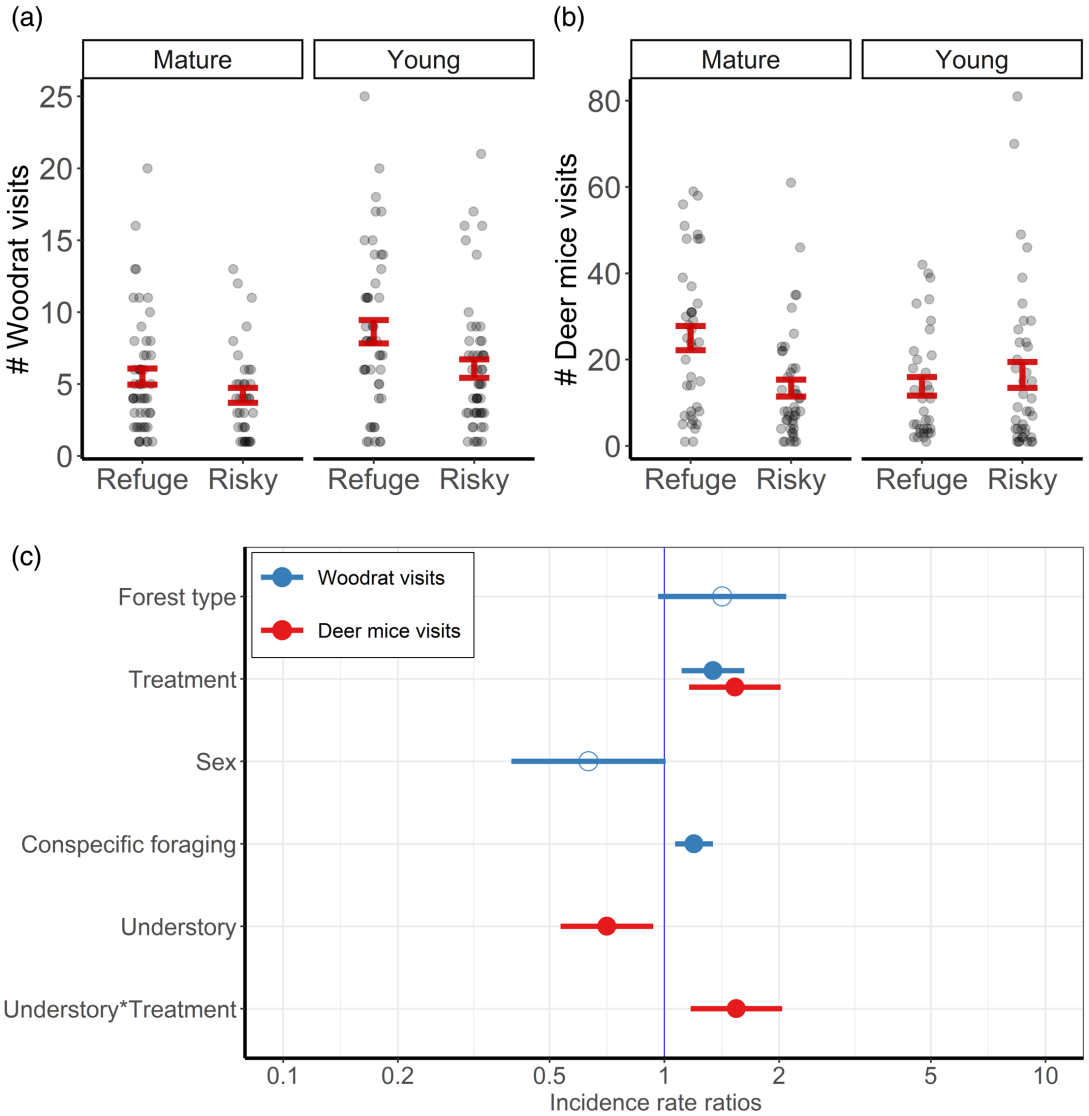
Number of nightly patch visits by dusky‐footed woodrats (*Neotoma fuscipes*) and deer mice (*Peromyscus* spp.). Figures represent raw values and relationships relative to forest type and refuge treatment for woodrats (a) and deer mice (b), and coefficient estimates (c) from each best‐supported negative binomial linear mixed‐effects model and their associated 95% confidence intervals with significance denoted by solid circles. The reference levels for categorical modalities are ‘mature’ for forest type, ‘risky’ for treatment, and ‘female’ for sex.

Woodrats visited patches more frequently in young versus mature forests (Figure 2a). Further, the number of nightly visits was greatest at safe patches in young forests, followed by similar visitation frequencies at risky patches in young forests and safe patches in mature forests; risky patches in mature forests were visited the least (Figure 2a). Deer mice most frequently visited safe patches in mature forests, with little difference between risky patches in mature forests and either treatment in young forests (Figure 2b). Among woodrats, the number of patch visits increased at refuge treatments (*β*_refuge_ = 0.29 [0.11, 0.48]) and with increasing conspecific PRT (*β*_nontarget_prt_ = 0.18 [0.07, 0.29]; Figure 2c; Table S2). For deer mice, visitation frequency increased at safe patches (*β*_refuge_ = 0.43 [0.15, 0.70]), while understory had opposite effects between treatments; specifically, visitation increased with increasing understory at safe patches (*β*_refuge:understory_ = 0.44 [0.16, 0.71]), but decreased at risky patches (*β*_risky:understory_ = −0.35 [−0.63, −0.07]; Figure 2c; Table S3).

**FIGURE 3:**
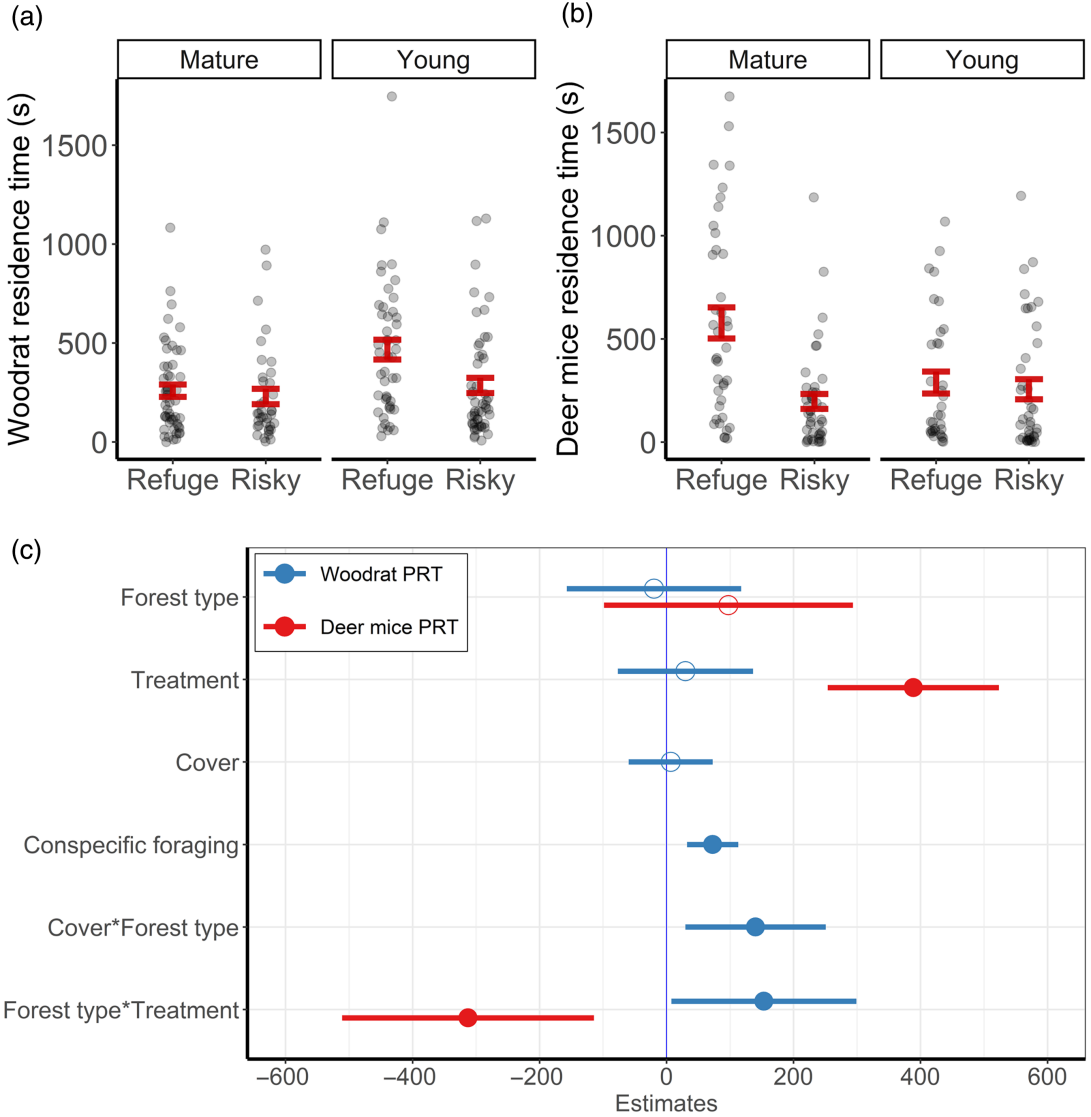
Cumulative patch residence time (PRT) by dusky‐footed woodrats (*Neotoma fuscipes*) and deer mice (*Peromyscus* spp.) during an experimental night. Figures represent raw values and relationships relative to forest type and refuge treatment for woodrats (a) and deer mice (b), and coefficient estimates (c) from each best‐supported linear mixed‐effects model and their associated 95% confidence intervals, with significance denoted by solid circles. The reference levels for categorical modalities are ‘mature’ for forest type and ‘risky’ for treatment.

Woodrats spent the most time in safe patches in young forests, with no significant difference in PRT between risky patches in young forests and either treatment in mature forests (Figure 3a). Deer mice spent nearly 3× longer at safe versus risky patches in mature forests, while there was no difference between treatments in young forests (Figure 3b). Among woodrats, PRT increased with conspecific PRT (*β*_nontarget_PRT_ = 72.65 [32.62, 112.69]), canopy cover within young forests (*β*_young:cover_ = 140.29 [30.53, 250.04]), and at safe patches within young forests (*β*_young:refuge_ = 153.29 [8.59, 297.99]; Figure 3c; Table S2). For deer mice, the top model for PRT only included the interaction between forest type and treatment, as individuals spent more time in safe patches within mature forests (*β*_mature:refuge_ = 388.81 [255.13, 522.48]), but less time in young forests (*β*_young:refuge_ = −312.50 [−509.15, −115.84]; Figure 3c; Table S3).

### 3.3 Stage 3: Patch behaviour

**FIGURE 4:**
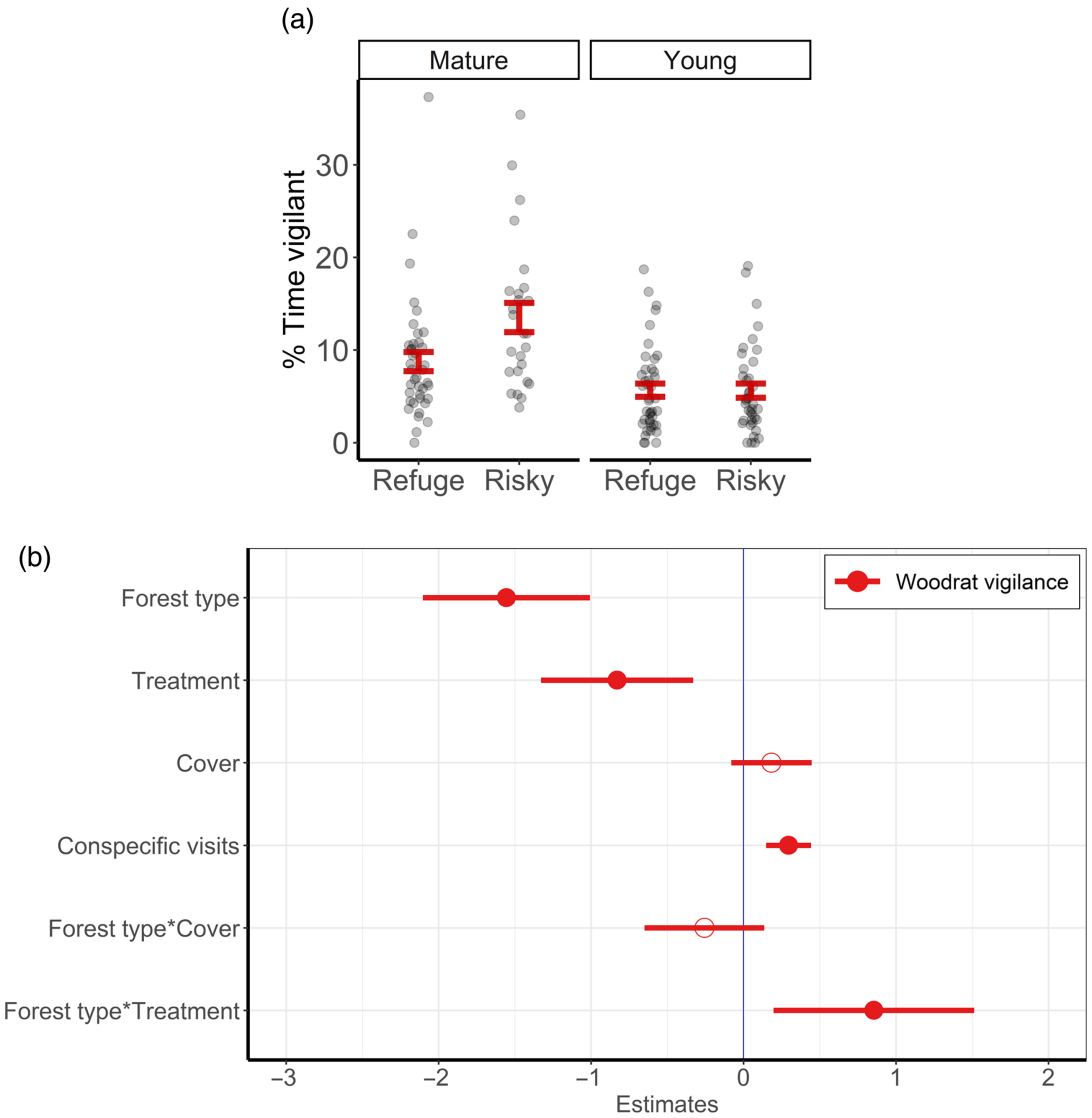
Percentage of nightly patch residence time allocated to vigilance behaviour among dusky‐footed woodrats (*Neotoma fuscipes*). Figures represent raw values and relationships relative to forest type and refuge treatment (a), and coefficient estimates (b) from the best‐supported linear mixed‐effects model and associated 95% confidence intervals with significance denoted by solid circles. The reference levels for categorical modalities are ‘mature’ for forest type and ‘risky’ for treatment.

Woodrats allocated almost twice as much proportional time to vigilance within mature versus young forests (*β*_young_ = −1.56 [−2.10, −1.01]; Figure 4a) and were more vigilant at risky versus safe patches in mature forests (*β*_mature:refuge_ = −0.83 [−1.32, −0.33]; Figure 4b; Table S2), but not young forests. Woodrats also increased vigilance with increasing frequency of patch visits by other woodrats (*β*_nontarget_visits_ = 0.30 [0.15, 0.44]).

### 3.4 Stage 4: Patch quitting

**FIGURE 5:**
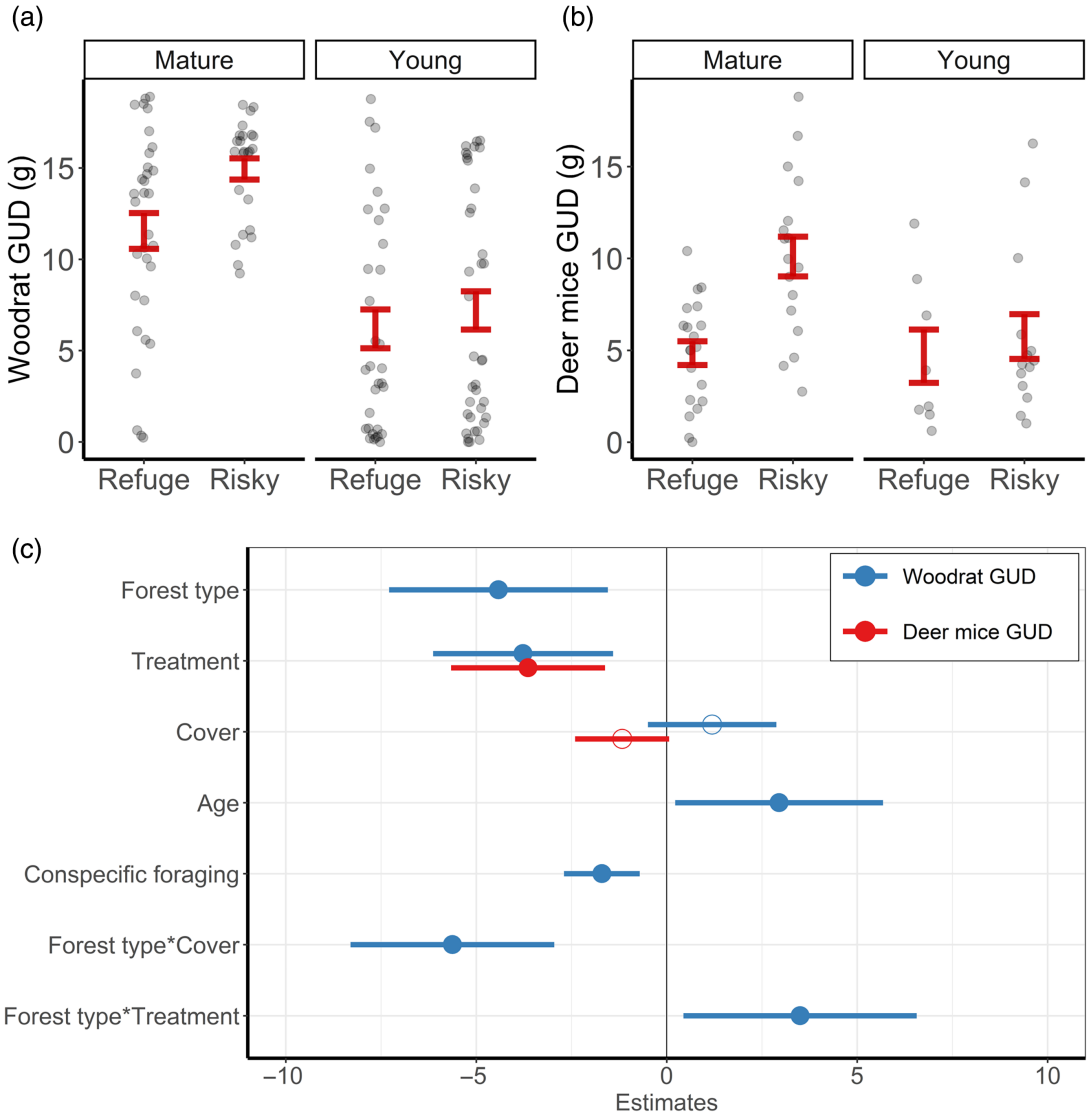
Giving‐up densities (GUDs) for dusky‐footed woodrats (*Neotoma fuscipes*) and deer mice (*Peromyscus* spp.). Figures represent raw values and relationships relative to forest type and refuge treatment for woodrats (a) and deer mice (b), and coefficient estimates (c) for both species from each best‐supported linear mixed‐effects model and their associated 95% confidence intervals with significance denoted by solid circles. The reference levels for categorical modalities are ‘mature’ for forest type, ‘risky’ for treatment, and ‘subadult’ for age.

Woodrat GUDs were double in mature versus young forests; further, woodrats exploited safe patches more within mature but not young forests (Figure 5a). Deer mice followed a similar pattern, as they foraged more at safe than risky patches in mature forests but not within young forests (Figure 5b). In addition to young forests (*β*_young_ = −4.42 [−7.26, −1.57]) and safe versus risky patches in mature forests (*β*_young:refuge_ = −3.77 [−6.11, −1.43]), woodrat GUDs decreased with increasing conspecific PRT (*β*_nontarget_prt_ = −1.70 [−2.69, −0.72]) and canopy cover within young forests (*β*_young:cover_ = −5.63 [−8.27, −2.98]; Figure 5c; Table S2). For deer mice, GUDs were lower at safe versus risky patches (*β*_refuge_ = −3.64 [−5.62, −1.67]; Figure 5c; Table S3).

### 3.5 Harvest rate curves

**FIGURE 6:**
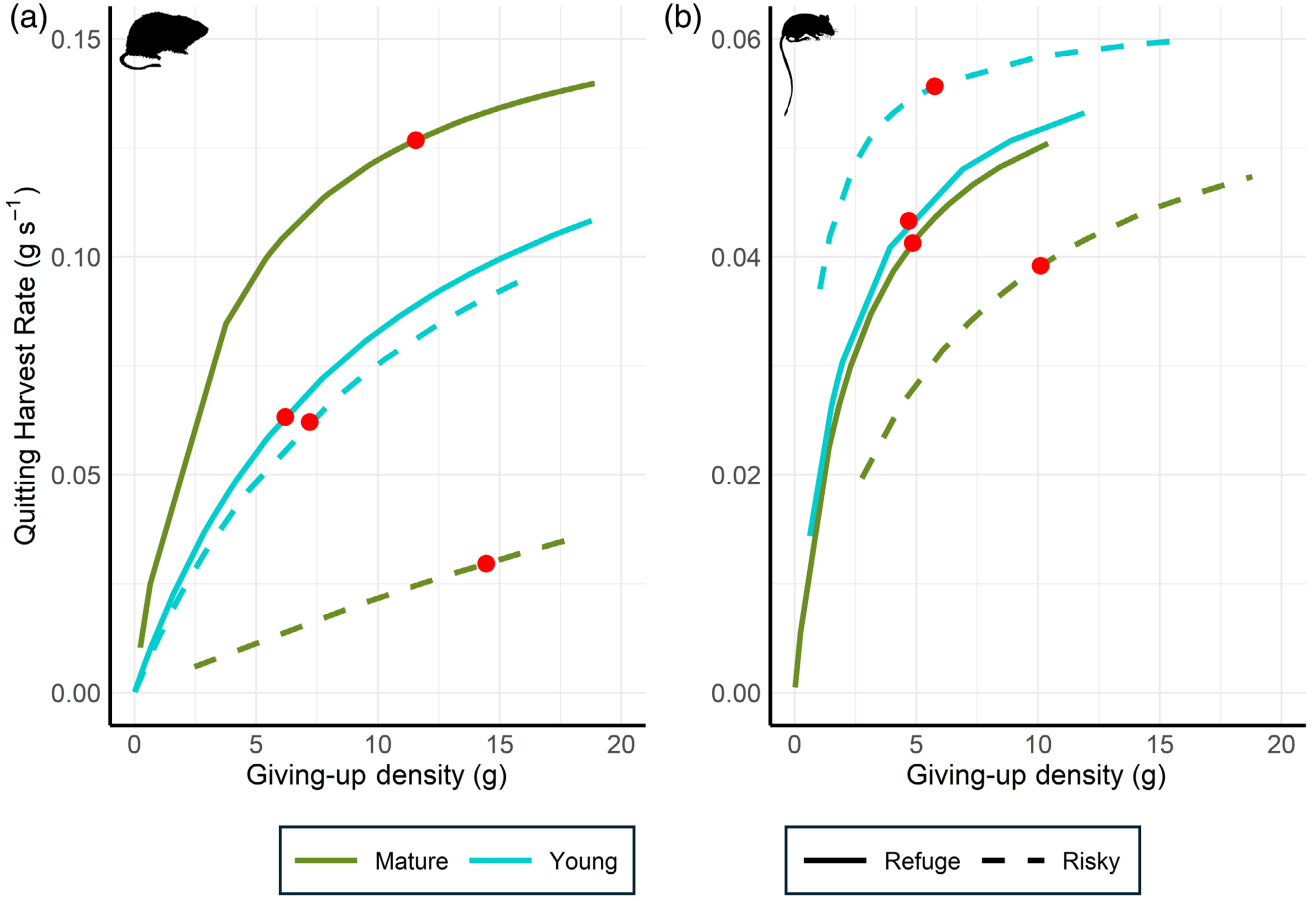
Harvest rate curves for dusky‐footed woodrats (*Neotoma fuscipes*; a) and deer mice (*Peromyscus* spp.; b) within four combinations of forest type and refuge treatment: Mature/refuge, mature/risky, young/refuge, and young/risky. Estimates of quitting harvest rates appear as functions of food density within the foraging patch. Curves are created by estimating attack rates and handling times from foraging data and fitting them to Holling's disc equation. For each combination, we plot the estimated quitting harvest rate (QHR) derived from GUD values and the disc equation. Red circles represent the characteristic QHR at mean GUD values for each group. Shallower slopes correspond with higher levels of vigilance; GUDs lying closer to the origin correspond with greater time allocation to foraging.

Woodrat QHRs were significantly different between forest type–refuge treatment combinations (*F*_3,134_ = 40.04, *p* < 0.0001). The QHR slope was steepest and the attack rate was highest at safe patches in mature forests (0.046 g s^−1^), followed by approximately equal slopes and attack rates at both safe (0.016 g s^−1^) and risky (0.014 g s^−1^) patches in young forests, with shallowest QHR slopes and lowest attack rates at risky patches in mature forests (0.003 g s^−1^; Figure 6a), indicating that the rate of harvest was quickest at safe patches in mature forests (i.e., apprehension is employed less as an antipredator strategy), while at mature‐risky combinations, woodrats foraged slowly and attentively. Accordingly, woodrats exhibited a characteristic QHR (i.e., QHR at mean GUD values) pattern of mature‐safe (0.127 g s^−1^) > young‐safe (0.063 g s^−1^) = young‐risky (0.062 g s^−1^) > mature‐risky (0.030 g s^−1^) and mean GUD pattern of mature‐risky (14.45 g) > mature‐safe (11.55 g) > young‐risky (7.20 g) > young‐safe (6.19 g).

Among deer mice, QHRs between forest type–refuge treatment combinations were also significantly different (*F*_3,78_ = 7.378, *p* < 0.001), yet followed different patterns than woodrats. QHR slopes were steepest and attack rates highest at safe patches in both young (0.030 g s^−1^) and mature (0.025 g s^−1^) forests, followed by considerably shallower slopes and lower attack rates at risky patches within mature (0.010 g s^−1^) and young (0.088 g s^−1^) forests (Figure 6b). Characteristic QHRs followed a different pattern of young‐risky (0.056 g s^−1^) > young‐safe (0.043 g s^−1^) = mature‐safe (0.040 g s^−1^) = mature‐risky (0.039 g s^−1^), and a mean GUD pattern of mature‐risky (10.10 g) > young‐risky (5.75 g) > mature‐safe (4.85 g) = young‐safe (4.68 g).

## 4 DISCUSSION

We found that asymmetrical predation on primary and secondary prey precipitated differences between the two species both in terms of risk perception and consequent strategies used to manage risk. Woodrats (primary prey) demonstrated behavioural responses to both forest type and patch treatment at each stage of the foraging process, while deer mice (alternative prey) only responded consistently to patch treatment. Moreover, while refuge treatments decreased perceived risk among deer mice regardless of forest type, for woodrats its importance was contingent upon the specific forest type in which it was located. These findings highlight how background risk mediates the relative perception of immediate risk and how these interactions can vary between primary and secondary prey.

Habitat structure is frequently used as a proxy for risk perception (Gaynor et al., 2019). Given that spotted owls predominantly forage within mature forests (Zulla et al., 2022), we expected that primary prey would perceive and respond to elevated risk within those areas. Throughout the foraging process, woodrats were more vigilant and foraged less within riskier mature forests and at refuge treatments within mature forests, but not within safer young forests. Together, these findings suggest that habitat characteristics that mediate immediate risk (e.g., protective cover or escape impediments) can interact with predator space use to ultimately shape perceived risk (Moll et al., 2017). In contrast, deer mice responded consistently to patch treatment but not forest type; further, among deer mice, the relative effect of refuge treatment was less dependent on the level of background risk than woodrats. We show that even within the same community featuring a shared predator, habitat features and predator activity may structure antipredator behaviours differently among primary and secondary prey. Therefore, the spatiotemporal pattern of risk experienced by prey is an emergent outcome between not only the properties of the predator or the setting in which the encounter may take place, but also the magnitude of predation relative to the overall prey community (Sheriff et al., 2020; Wirsing et al., 2021).

Consumptive effects of predators on primary and secondary prey have received considerable attention in ecological research (Holt & Lawton, 1994). Predator‐mediated apparent competition can yield various outcomes, from competitor exclusion to increased predator densities (Bonsall & Hassell, 1997; Wilson et al., 2022). In systems with a clear prey hierarchy, targeted consumption of primary prey can relieve secondary prey from predation pressure, increasing secondary prey abundance while simultaneously decreasing overall prey biomass (Holt & Lawton, 1994; Moran & Hurd, 1997). A small number of studies have linked antipredator behaviour to factors such as the effectiveness of risk management strategies (Dellinger et al., 2019), community composition (Prasad & Snyder, 2006), or baseline risk prior to the addition of a novel predator (Makin et al., 2018). However, nonconsumptive effects within multiprey systems, particularly within the paradigm of primary and secondary prey, still remain largely overlooked. The interactions between consumptive and nonconsumptive effects can vary in strength and nature (Matassa & Trussell, 2011) and increases in perceived risk can drive countervailing effects between lethal and nonlethal impacts (Prasad & Snyder, 2006). We found that primary—but not secondary—prey exhibit stronger antipredator behaviours that closely mirror actual patterns of risk. Therefore, in addition to experiencing stronger consumptive effects, our findings suggest that primary prey invest more in antipredator strategies than secondary prey, resulting in greater nonconsumptive effects, particularly when background risk is high. Thus, the demographic consequences of nonconsumptive effects may also indirectly benefit alternative prey by decreasing competitor abundance in a manner similar to consumptive effects (Laundré et al., 2014; Moran & Hurd, 1997). Nevertheless, there may also be consequences for alternative prey. Asymmetrical and spatially accurate risk management by primary prey can indirectly affect the community by reducing the ratio of available prey and thereby increasing relative predation pressure on naive secondary species (Bonsall & Hassell, 1997). We contrasted the benefit of risk dilution and competitive release with the potential cost of enemy‐mediated competition by testing whether the presence of interspecific foragers modulated perceived risk. However, none of our variables (i.e., presence, number of visits, or PRT) affected any stage of the foraging process for either woodrats or deer mice. In contrast, among woodrats conspecific activity influenced nearly every stage of the foraging process, underscoring the effect of group foraging on perceived risk and the value of a resource patch (Alexander, 1974; Carthey & Banks, 2015). While the loss or addition of species can have far‐reaching trophic effects (Holt & Lawton, 1994)—and in some invertebrate systems may also have non‐trophic effects (Moran & Hurd, 1997; Steffan & Snyder, 2010)—we did not find evidence for this in our system. Therefore, asymmetrical risk management may come at a cost to primary prey by elevating nonconsumptive effects with little to no net benefit to predators or secondary prey.

Beyond predation intensity, our study highlights additional drivers of NCE contingencies that arise from properties of predator and prey (Schmitz, 2007), especially predator hunting mode and the relative overlap of habitat domains between predator and prey (Gaynor et al., 2019; Schmitz et al., 2017). When prey are mobile and have broad domains, they typically select predator‐free spatial refuge to minimize the likelihood of encounter (Dellinger et al., 2019; Wirsing et al., 2021). Conversely, when predator hunting domains overlap or exceed those of their prey—as with spotted owls—prey must rely more on behavioural adjustments and use local space in a manner that facilitates their evasion strategies (Schmitz et al., 2017). Predator hunting mode (e.g., active versus ambush predation) can also influence risk management strategies (Makin et al., 2017; Preisser et al., 2007). Ambush predators, including owls, have a relatively continuous and spatially predictable presence (Brown, 1999; Zulla et al., 2022), which creates a heterogeneous pattern of background risk across their home range (Gaynor et al., 2019). While other predator species were present, our study system is relatively simple in terms of those that could theoretically consume woodrats or deer mice and we do not believe this affected our results for several reasons. First, woodrats and deer mice are nocturnal species (Kelt et al., 2017; Sakai & Noon, 1997), which eliminates the potential effect of diurnal species and all avian predators besides great horned owls (*Bubo virginianus*), which have a generalist diet containing far fewer woodrats than spotted owls (Bogiatto et al., 2003; Maser et al., 1970). Further, the diversity of potential mammalian predators in this system is limited to grey foxes, coyotes (*Canis latrans*), and long‐tailed weasels (*Mustela frenata*), each of which likely had a negligible effect on risk perception as (A) prior work in this system found low woodrat mortalities relative to mammalian predators (Kuntze et al., 2023), (B) we only detected foxes at foraging stations during four total tray‐nights with no detections of other predators, and (C) we designed our refuge treatment to mediate the threat of vertical predation specifically. Indeed, studies on small mammals have found that foraging under protective cover may reduce the risk of avian predation (Verdolin, 2006), but simultaneously increase the risk of predation from snakes or mammals (Embar et al., 2011). This may explain why the presence of cover at a patch was only important in mature forests among woodrats. The decision to forage more at the ‘safe’ patch within ostensibly ‘riskier’ mature forest but not within a young forest suggests that from a woodrat's perspective, refuge treatments may represent islands of relative safety in a sea of risky forest (Embar et al., 2014). In contrast, for deer mice, changes in antipredator behaviour relative to refuge treatment but not forest type suggest that outside of the immediate vicinity, perceived risk is relatively homogeneous by forest type and agnostic of the actual risk of encounter.

Evaluating the specific behavioural tools used to manage risk can improve our understanding of risk perception beyond GUDs and foraging time in the context of how organisms resolve the trade‐off between food and safety. Indeed, while the foraging cost of predation is a product of the magnitude of risk and the forager's state (Kotler et al., 2010), the strategies used to manage it can vary by species, sensory modality, or even the relative investment in one strategy over another (Kotler et al., 2010; Wirsing et al., 2021). By quantifying the relationship between foraging success and resource density, the slope and shape of the harvest curve illustrate how prey alter their use of time allocation and vigilance to manage risk under varying conditions (Brown, 1999; Kotler et al., 2010). Through these, we show that within young forests woodrats use a similar combination of strategies to manage risk regardless of refuge treatment at the foraging patch. Yet, while woodrats responded to increased risk in mature forests with a lower average harvest, the manner by which they reached higher GUDs differed between refuge treatments. Specifically, woodrats at risky patches predominantly used vigilance to manage risk—as evidenced by shallower slopes—while at safe patches they mostly abandoned vigilance—resulting in steeper slopes. As a result, while time allocation was comparable between refuge treatments, at safe patches, the QHR was higher and GUD was lower. These reveal a nuanced response wherein vigilance is used to manage risk within ‘risky’ environments but is largely abandoned in the presence of a ‘safe’ refuge treatment. This may be a result of vigilance no longer being effective or necessary as the presence of vertical cover reduces the acute risk of avian predation (Embar et al., 2011; Verdolin, 2006). In contrast, deer mice had comparable slopes and mean GUD values at safe treatments regardless of forest type, while at risky patches deer mice were equally vigilant between forest types yet differed in time allocation—which produced similar slopes but higher mean GUD values in mature forests. Taken together, these results demonstrate a behavioural approach wherein vigilance and time allocation are used to manage risk by both primary and secondary prey, but in different manners and at times, opposing circumstances. For woodrats, our findings align with previous studies that imply vigilance is used more in locations where background risk is high and when acute risk is also high, but not when acute risk is low (Dröge et al., 2017; Embar et al., 2011). In contrast, deer mice used vigilance more where acute risk was high, independent of background risk. These results also then suggest that primary prey are responsive to and manage both background and acute threats whereas secondary prey primarily rely upon managing acute threats to mitigate risk.

Our findings provide some of the first empirical evidence that among a diverse prey guild within a spatially heterogeneous environment, asymmetrical predation can modulate not only the magnitude of antipredator behaviours but also the specific strategies used to manage risk. These behavioural differences between primary and secondary prey have implications for both the species involved as well as the community when contrasted with the direct effects of asymmetrical predation. Consumptive effects impose costs only on those animals actually consumed, which translate to direct benefits for the predator (Ives & Dobson, 1987), while the costs of NCEs are paid by the entire prey population and do not benefit (or lead to more) predators (Ives & Dobson, 1987; Wirsing et al., 2021). Further, antipredator behaviours produce feedbacks in predation rates and the predator population more rapidly than feedback from direct consumption (Ives & Dobson, 1987), which may stabilize oscillations in predator and prey densities (Laundré, 2010). The presence of multiple prey species—particularly when one is disproportionately targeted—adds additional complexity to these dynamics. While primary prey experience greater consumptive effects (i.e., per‐capita mortality rates) than secondary prey, we show that the relative difference in nonconsumptive effects between the two is even greater when background risk is high, yet reduced when background risk is low. Thus, if risk‐induced fitness consequences have an additive rather than compensatory effect on prey, these differences can alter population size, and in turn, community assemblages (Donadio & Buskirk, 2016). Amidst a growing literature of NCEs, there is a drive to understand how risk influences population size and the manner in which species are represented in a given environment (Sheriff et al., 2020). Here, we highlight an important and understudied contingency in how predation risk effects among prey may extend to population‐ and community‐level responses.

These differences may have emergent consequences for predator populations as well. Optimal foraging theory suggests that a predator should select the most beneficial prey in terms of net energy gain relative to searching and handling time (Stephens & Krebs, 1986). When primary prey exhibit stronger antipredator behaviour that accurately reflects background risk, the trade‐off between biomass and naivete may influence patterns in predator dietary specialization. Spotted owls benefit from selecting prey that most efficiently balances foraging costs with energetic returns (Hobart et al., 2019; Stephens & Krebs, 1986). As such, woodrats often dominate owl diets in occurrence and biomass (Hobart et al., 2019), although in many parts of their range owls still consume a sizable number of alternative prey including deer mice (Kuntze et al., 2023; Zulla et al., 2022). Increasing consumption of larger‐bodied woodrats has emergent benefits for spotted owl occupancy and fitness (Hobart et al., 2019; Kuntze et al., 2023). However, overreliance on risk‐averse species may increase energetic expenditures associated with prey searching, particularly when primary prey abundance is low (Balme et al., 2020; Ives & Dobson, 1987), highlighting a potential benefit of consuming naive deer mice. Asymmetrical predation is not uncommon among prey guilds with shared predators (Holt & Lawton, 1994). Therefore, these findings have conservation implications for both predators and prey beyond the ones studied here. While high‐quality prey is typically the prominent driver of population dynamics among relatively specialized predators (Hobart et al., 2019; Kuntze et al., 2023), we suggest that the naivete of alternative prey may allow individuals to better exploit this resource base and meet energetic demands during periods of low primary prey availability or within highly impacted ecosystems (Balme et al., 2020). Evaluating consumptive and nonconsumptive effects through the paradigm of primary and secondary prey may advance predator conservation as well as our understanding of how NCEs propagate through complex communities.

## AUTHOR CONTRIBUTIONS

Corbin C. Kuntze, Jonathan N. Pauli, and M. Zachariah Peery conceived the ideas and designed methodology; Corbin C. Kuntze collected the data; Corbin C. Kuntze and Jonathan N. Pauli analysed the data and led the writing of the manuscript, with key input from M. Zachariah Peery. All authors contributed critically to the drafts and gave final approval for publication.

## CONFLICT OF INTEREST STATEMENT

The authors declare no conflict of interest.

## Supporting information


**Appendix S1:** Supplementary methods.
**Appendix S2:** Supplementary results.

## Data Availability

Data available from the Dryad Digital Repository: https://doi.org/10.5061/dryad.qjq2bvqqp (Kuntze et al., 2024).

## References

[jane14166-bib-0001] Alexander, R. D. (1974). The evolution of social behavior. Annual Review of Ecology and Systematics, 5, 325–383.

[jane14166-bib-0002] Balme, G. A. , le Roex, N. , Rogan, M. S. , & Hunter, L. T. B. (2020). Ecological opportunity drives individual dietary specialization in leopards. Journal of Animal Ecology, 89(2), 589–600. 31579935 10.1111/1365-2656.13109

[jane14166-bib-0064] Bedoya‐Perez, M. A. , Carthey, A. J. R. , Mella, V. S. A. , McArthur, C. , & Banks, P. B. (2013). A practical guide to avoid giving up on giving‐up densities. Behavioral Ecology and Sociobiology, 67, 1541–1553.

[jane14166-bib-0003] Bogiatto, R. J. , Sardella, B. A. , & Essex, J. J. (2003). Food habits of great horned owls in northeastern California with notes on seasonal diet shifts. Western North American Naturalist, 63(2), 258–263.

[jane14166-bib-0004] Bonsall, M. B. , & Hassell, M. P. (1997). Apparent competition structures ecological assemblages. Nature, 388(6640), 371–373.

[jane14166-bib-0065] Brown, J. S. (1988). Patch use as an indicator of habitat preference, predation risk, and competition. Behavioral Ecology and Sociobiology, 22, 37–47.

[jane14166-bib-0005] Brown, J. S. (1999). Vigilance, patch use and habitat selection: Foraging under predation risk. Evolutionary Ecology Research, 1(1), 49–71.

[jane14166-bib-0006] Brown, J. S. , & Kotler, B. P. (2004). Hazardous duty pay and the foraging cost of predation. Ecology Letters, 7(10), 999–1014.

[jane14166-bib-0007] Brown, J. S. , Kotler, B. P. , & Bouskila, A. (2001). Ecology of fear: Foraging games between predators and prey with pulsed resources. Annales Zoologici Fennici, 38, 71–87.

[jane14166-bib-0008] Carthey, A. J. R. , & Banks, P. B. (2015). Foraging in groups affects giving‐up densities: Solo foragers quit sooner. Oecologia, 178(3), 707–713. 25740332 10.1007/s00442-015-3274-x

[jane14166-bib-0009] Creel, S. (2011). Toward a predictive theory of risk effects: Hypotheses for prey attributes and compensatory mortality. Ecology, 92(12), 2190–2195. 22352157 10.1890/11-0327.1

[jane14166-bib-0010] Dellinger, J. A. , Shores, C. R. , Craig, A. , Heithaus, M. R. , Ripple, W. J. , & Wirsing, A. J. (2019). Habitat use of sympatric prey suggests divergent anti‐predator responses to recolonizing gray wolves. Oecologia, 189(2), 487–500. 30539299 10.1007/s00442-018-4323-z

[jane14166-bib-0011] Donadio, E. , & Buskirk, S. W. (2016). Linking predation risk, ungulate antipredator responses, and patterns of vegetation in the high Andes. Journal of Mammalogy, 97(3), 966–977.

[jane14166-bib-0012] Dormann, C. F. , Elith, J. , Bacher, S. , Buchmann, C. , Carl, G. , Carré, G. , Marquéz, J. R. G. , Gruber, B. , Lafourcade, B. , & Leitão, P. J. (2013). Collinearity: A review of methods to deal with it and a simulation study evaluating their performance. Ecography, 36(1), 27–46.

[jane14166-bib-0013] Dröge, E. , Creel, S. , Becker, M. S. , & M'soka, J. (2017). Spatial and temporal avoidance of risk within a large carnivore guild. Ecology and Evolution, 7(1), 189–199. 28070283 10.1002/ece3.2616 PMC5215178

[jane14166-bib-0014] Embar, K. , Kotler, B. P. , & Mukherjee, S. (2011). Risk management in optimal foragers: The effect of sightlines and predator type on patch use, time allocation, and vigilance in gerbils. Oikos, 120(11), 1657–1666.

[jane14166-bib-0015] Embar, K. , Raveh, A. , Hoffmann, I. , & Kotler, B. P. (2014). Predator facilitation or interference: A game of vipers and owls. Oecologia, 174(4), 1301–1309. 24481981 10.1007/s00442-013-2760-2

[jane14166-bib-0016] Fraik, A. K. , Facka, A. N. , & Powell, R. A. (2023). Food and cover resources for small mammals on an industrially logged landscape in the Sierra Nevada of California. Canadian Journal of Zoology, 101, 473–485.

[jane14166-bib-0017] Friard, O. , & Gamba, M. (2016). BORIS: A free, versatile open‐source event‐logging software for video/audio coding and live observations. Methods in Ecology and Evolution, 7(11), 1325–1330.

[jane14166-bib-0018] Gaynor, K. M. , Brown, J. S. , Middleton, A. D. , Power, M. E. , & Brashares, J. S. (2019). Landscapes of fear: Spatial patterns of risk perception and response. Trends in Ecology & Evolution, 34(4), 355–368. 30745252 10.1016/j.tree.2019.01.004

[jane14166-bib-0019] Hobart, B. K. , Jones, G. M. , Roberts, K. N. , Dotters, B. P. , Whitmore, S. A. , Berigan, W. J. , Raphael, M. G. , Keane, J. J. , Gutiérrez, R. J. , & Peery, M. Z. (2019). Trophic interactions mediate the response of predator populations to habitat change. Biological Conservation, 238, 108217.

[jane14166-bib-0020] Holt, R. D. , & Lawton, J. H. (1994). The ecological consequences of shared natural enemies. Annual Review of Ecology and Systematics, 25, 495–520.

[jane14166-bib-0021] Ives, A. R. , & Dobson, A. P. (1987). Antipredator behavior and the population dynamics of simple predator‐prey systems. The American Naturalist, 130(3), 431–447.

[jane14166-bib-0022] Jones, G. M. , Keane, J. J. , Gutiérrez, R. J. , & Peery, M. Z. (2018). Declining old‐forest species as a legacy of large trees lost. Diversity and Distributions, 24(3), 341–351.

[jane14166-bib-0023] Kachel, S. , Bayrakcısmith, R. , Kubanychbekov, Z. , Kulenbekov, R. , McCarthy, T. , Weckworth, B. , & Wirsing, A. (2023). Ungulate spatiotemporal responses to contrasting predation risk from wolves and snow leopards. Journal of Animal Ecology, 92(1), 142–157. 36416593 10.1111/1365-2656.13850

[jane14166-bib-0024] Kelt, D. A. , Sollmann, R. , White, A. M. , Roberts, S. L. , & Van Vuren, D. H. (2017). Diversity of small mammals in the Sierra Nevada: Filtering by natural selection or by anthropogenic activities? Journal of Mammalogy, 98(1), 85–93.

[jane14166-bib-0025] Kohl, M. T. , Ruth, T. K. , Metz, M. C. , Stahler, D. R. , Smith, D. W. , White, P. J. , & MacNulty, D. R. (2019). Do prey select for vacant hunting domains to minimize a multi‐predator threat? Ecology Letters, 22(11), 1724–1733. 31373137 10.1111/ele.13319

[jane14166-bib-0026] Kotler, B. P. (2016). Fun and games: Predator‐prey foraging games and related interactions. Israel Journal of Ecology & Evolution, 62(3–4), 118–120.

[jane14166-bib-0027] Kotler, B. P. , & Blaustein, L. (1995). Titrating food and safety in a heterogeneous environment: When are the risky and safe patches of equal value? Oikos, 74, 251–258.

[jane14166-bib-0028] Kotler, B. P. , Brown, J. , Mukherjee, S. , Berger‐Tal, O. , & Bouskila, A. (2010). Moonlight avoidance in gerbils reveals a sophisticated interplay among time allocation, vigilance and state‐dependent foraging. Proceedings of the Royal Society B: Biological Sciences, 277(1687), 1469–1474. 10.1098/rspb.2009.2036 PMC2871830 20053649

[jane14166-bib-0029] Kotler, B. P. , & Brown, J. S. (1990). Rates of seed harvest by two species of gerbilline rodents. Journal of Mammalogy, 71(4), 591–596.

[jane14166-bib-0030] Kuntze, C. C. , Pauli, J. N. , Zulla, C. J. , Keane, J. J. , Roberts, K. N. , Dotters, B. P. , Sawyer, S. C. , & Peery, M. Z. (2023). Landscape heterogeneity provides co‐benefits to predator and prey. Ecological Applications, 33(8), e2908. 37602901 10.1002/eap.2908

[jane14166-bib-0031] Kuntze, C. C. , Peery, M. Z. , & Pauli, J. N. (2024). Data from: Asymmetrical predation intensity produces divergent antipredator behaviors in primary and secondary prey. *Dryad Digital Repository*, 10.5061/dryad.qjq2bvqqp PMC11615267 39205404

[jane14166-bib-0032] Laundré, J. W. (2010). Behavioral response races, predator‐prey shell games, ecology of fear, and patch use of pumas and their ungulate prey. Ecology, 91(10), 2995–3007. 21058559 10.1890/08-2345.1

[jane14166-bib-0033] Laundré, J. W. , Hernández, L. , Medina, P. L. , Campanella, A. , López‐Portillo, J. , González‐Romero, A. , Grajales‐Tam, K. M. , Burke, A. M. , Gronemeyer, P. , & Browning, D. M. (2014). The landscape of fear: The missing link to understand top‐down and bottom‐up controls of prey abundance? Ecology, 95(5), 1141–1152. 25000746 10.1890/13-1083.1

[jane14166-bib-0034] Lima, S. L. , & Bednekoff, P. A. (1999). Temporal variation in danger drives antipredator behavior: The predation risk allocation hypothesis. The American Naturalist, 153(6), 649–659. 10.1086/303202 29585647

[jane14166-bib-0035] Lima, S. L. , & Dill, L. M. (1990). Behavioral decisions made under the risk of predation: A review and prospectus. Canadian Journal of Zoology, 68(4), 619–640.

[jane14166-bib-0036] Makin, D. F. , Chamaillé‐Jammes, S. , & Shrader, A. M. (2017). Herbivores employ a suite of antipredator behaviours to minimize risk from ambush and cursorial predators. Animal Behaviour, 127, 225–231.

[jane14166-bib-0037] Makin, D. F. , Chamaillé‐Jammes, S. , & Shrader, A. M. (2018). Changes in feeding behavior and patch use by herbivores in response to the introduction of a new predator. Journal of Mammalogy, 99(2), 341–350.

[jane14166-bib-0038] Maser, C. , Hammer, E. W. , & Anderson, S. H. (1970). Comparative food habits of three owl species in Central Oregon. The Murrelet, 51(3), 29–33.

[jane14166-bib-0039] Matassa, C. M. , & Trussell, G. C. (2011). Landscape of fear influences the relative importance of consumptive and nonconsumptive predator effects. Ecology, 92(12), 2258–2266. 22352165 10.1890/11-0424.1

[jane14166-bib-0040] Moll, R. J. , Redilla, K. M. , Mudumba, T. , Muneza, A. B. , Gray, S. M. , Abade, L. , Hayward, M. W. , Millspaugh, J. J. , & Montgomery, R. A. (2017). The many faces of fear: A synthesis of the methodological variation in characterizing predation risk. Journal of Animal Ecology, 86(4), 749–765. 28390066 10.1111/1365-2656.12680

[jane14166-bib-0041] Moran, M. D. , & Hurd, L. E. (1997). A trophic cascade in a diverse arthropod community caused by a generalist arthropod predator. Oecologia, 113, 126–132. 28307287 10.1007/s004420050360

[jane14166-bib-0042] Morin, D. J. , Yackulic, C. B. , Diffendorfer, J. E. , Lesmeister, D. B. , Nielsen, C. K. , Reid, J. , & Schauber, E. M. (2020). Is your ad hoc model selection strategy affecting your multimodel inference? Ecosphere, 11(1), e02997.

[jane14166-bib-0043] Prasad, R. P. , & Snyder, W. E. (2006). Polyphagy complicates conservation biological control that targets generalist predators. Journal of Applied Ecology, 43, 343–352.

[jane14166-bib-0044] Preisser, E. L. , Bolnick, D. I. , & Benard, M. F. (2005). Scared to death? The effects of intimidation and consumption in predator‐prey interactions. Ecology, 86(2), 501–509.

[jane14166-bib-0045] Preisser, E. L. , Orrock, J. L. , & Schmitz, O. J. (2007). Predator hunting mode and habitat domain alter nonconsumptive effects in predator‐prey interactions. Ecology, 88(11), 2744–2751. 18051642 10.1890/07-0260.1

[jane14166-bib-0046] Prugh, L. R. , & Golden, C. D. (2014). Does moonlight increase predation risk? Meta‐analysis reveals divergent responses of nocturnal mammals to lunar cycles. Journal of Animal Ecology, 83(2), 504–514. 24102189 10.1111/1365-2656.12148

[jane14166-bib-0047] R Core Team . (2023). R: A language and environment for statistical computing [computer software]. R Foundation for Statistical Computing.

[jane14166-bib-0048] Sakai, H. F. , & Noon, B. R. (1993). Dusky‐footed woodrat abundance in different‐aged forests in northwestern California. The Journal of Wildlife Management, 57(2), 373.

[jane14166-bib-0049] Sakai, H. F. , & Noon, B. R. (1997). Between‐habitat movement of dusky‐footed woodrats and vulnerability to predation. The Journal of Wildlife Management, 61(2), 343.

[jane14166-bib-0050] Schmitz, O. J. (2007). Predator diversity and trophic interactions. Ecology, 88(10), 2415–2426. 18027743 10.1890/06-0937.1

[jane14166-bib-0051] Schmitz, O. J. , Miller, J. R. B. , Trainor, A. M. , & Abrahms, B. (2017). Toward a community ecology of landscapes: Predicting multiple predator‐prey interactions across geographic space. Ecology, 98(9), 2281–2292. 28585719 10.1002/ecy.1916

[jane14166-bib-0052] Schmitz, O. J. , & Trussell, G. C. (2016). Multiple stressors, state‐dependence and predation risk—Foraging trade‐offs: Toward a modern concept of trait‐mediated indirect effects in communities and ecosystems. Current Opinion in Behavioral Sciences, 12, 6–11.

[jane14166-bib-0053] Schulte‐Hostedde, A. I. , Zinner, B. , Millar, J. S. , & Hickling, G. J. (2005). Restitution of mass‐size residuals: Validating body condition indices. Ecology, 86(1), 155–163.

[jane14166-bib-0054] Sheriff, M. J. , Peacor, S. D. , Hawlena, D. , & Thaker, M. (2020). Non‐consumptive predator effects on prey population size: A dearth of evidence. Journal of Animal Ecology, 89(6), 1302–1316. 32215909 10.1111/1365-2656.13213

[jane14166-bib-0055] Sikes, R. S. , & the Animal Care and Use Committee of the American Society of Mammalogists . (2016). 2016 guidelines of the American Society of Mammalogists for the use of wild mammals in research and education. Journal of Mammalogy, 97, 663–688. 29692469 10.1093/jmammal/gyw078 PMC5909806

[jane14166-bib-0056] Steffan, S. A. , & Snyder, W. E. (2010). Cascading diversity effects transmitted exclusively by behavioral interactions. Ecology, 91(8), 2242–2252. 20836446 10.1890/09-0787.1

[jane14166-bib-0057] Stephens, D. W. , & Krebs, J. R. (1986). Foraging theory. Princeton University Press.

[jane14166-bib-0058] Verdolin, J. L. (2006). Meta‐analysis of foraging and predation risk trade‐offs in terrestrial systems. Behavioral Ecology and Sociobiology, 60(4), 457–464.

[jane14166-bib-0059] Wilson, E. C. , Zuckerberg, B. , Peery, M. Z. , & Pauli, J. N. (2022). Experimental repatriation of snowshoe hares along a southern range boundary reveals historical community interactions. Ecological Monographs, 92(3), e1509.

[jane14166-bib-0060] Wirsing, A. J. , Cameron, K. E. , & Heithaus, M. R. (2010). Spatial responses to predators vary with prey escape mode. Animal Behaviour, 79(3), 531–537.

[jane14166-bib-0061] Wirsing, A. J. , Heithaus, M. R. , Brown, J. S. , Kotler, B. P. , & Schmitz, O. J. (2021). The context dependence of non‐consumptive predator effects. Ecology Letters, 24(1), 113–129. 32990363 10.1111/ele.13614

[jane14166-bib-0062] Wolf, N. , & Mangel, M. (2007). Strategy, compromise, and cheating in predator‐prey games. Evolutionary Ecology Research, 9(8), 1293–1304.

[jane14166-bib-0063] Zulla, C. J. , Kramer, H. A. , Jones, G. M. , Keane, J. J. , Roberts, K. N. , Dotters, B. P. , Sawyer, S. C. , Whitmore, S. A. , Berigan, W. J. , Kelly, K. G. , Wray, A. K. , & Peery, M. Z. (2022). Large trees and forest heterogeneity facilitate prey capture by California spotted owls. Ornithological Applications, 124(3), duac024.

