# Supplementary material for: Asymmetrical predation intensity produces divergent antipredator behaviours in primary and secondary prey
Source: J Anim Ecol. 2024 Aug 28;93(12):2038–50. doi: 10.1111/1365-2656.14166 (PMC11615267; doi:10.1111/1365-2656.14166)
Supplement: Supplementary file 1 — Appendix S1: Supplementary methods. Appendix S2: Supplementary results. [file JANE-93-2038-s001.docx]

Journal of Animal Ecology

Supplementary Materials

**Asymmetrical predation intensity produces divergent antipredator behaviors in primary and secondary prey**

**Appendix S1 – Supplementary methods**

***Field methods for capturing and monitoring woodrats***

We captured woodrats in steel mesh traps (model #105; Tomahawk Live Trap Company, Hazelhurst, Wisconsin, USA) baited with a mix of birdseed, dried fruit, and peanuts. Individuals were ear punched and marked with a unique ear tag (Model 1005-1; National Band & Tag Company, Newport, KY) or passive integrated transponder (PIT) tag (Avid Identification Systems, Norco, CA). A subsample of these individuals (*n* = 73) weighing above 120g were fit with VHF collars (Lotek model TW-5, 10g; Lotek Wireless Inc, Newmarket, Ontario, Canada or Telenax model TXE-116C, 6g; Titley Scientific, Columbia, Missouri, USA) equipped with onboard activity sensors, which allowed us to monitor movement patterns and detect mortality events shortly after they occurred. All captures were done with approval by the Institutional Animal Care and Use Committee of the University of Wisconsin, Madison (IACUC #A006173-A01), and followed guidelines from the American Society of Mammalogists (Sikes et al. 2016).

***Additional detail for foraging station design***

Trays were not washed between nights throughout deployment at a specific foraging station (i.e., the same tray was used for the two acclimation and four data collection days) but were rinsed and typically left empty and unused for several days before moving to a new location and target individual. If a tray was disturbed or seemed unusual the sand matrix was replaced with a fresh matrix of sand. Otherwise, while sand was typically re-used in foraging patches, it was thoroughly sieved during each morning when the remaining food was collected from the matrix – so if any feces or particulate was present it would have been removed before the next night of data collection. However, neither species would typically urinate or defecate in the trays. There was little to no evidence of this occurring in our video data, and feces was rarely recovered (and at very low quantities) when the sand matrix was sieved for data collection in the morning following a foraging night.

Sikes, R. S. and the Animal Care and Use Committee of the American Society of Mammalogists. 2016. 2016 Guidelines of the American Society of Mammalogists for the use of wild mammals in research and education. Journal of Mammalogy 97:663–688.

| **Table S1.** Model covariates used for quantifying foraging behavior of dusky-footed woodrats (*Neotoma fuscipes*) and deer mice (*Peromyscus* spp.) in the Sierra Nevada, California, USA. | | |
| --- | --- | --- |
| **Category** | **Variable** | **Description** |
| Habitat | Forest type | Forest structure designation defined by canopy cover and quadratic mean diameter of dominant trees (mature, young) |
|  | Treatment | Presence/absence of artificial cover structure at foraging patch (risky, refuge) |
|  | Canopy cover | Proportion of sky obscured by canopy >2m height within 12.5m |
|  | Basal area | Total basal area (m^2^) of all live and dead standing trees <2m height within 12.5m |
|  | Understory | Proportion of ground obscured by vegetation <2m height within 12.5m |
| Intrinsic (Woodrat only) | Body condition | Residuals from the regression of body mass against hind foot length; positive values represent individuals in better body condition while negative values represent poor body condition |
|  | Age | Age class of target individual (adult, subadult) |
|  | Sex | Sex of target individual (female, male) |
| Other foragers (Stage 1-4) | Conspecifics | Presence/absence of additional woodrats beyond the target individual (yes, no; woodrat only) |
|  | Woodrat | Presence/absence of woodrats (yes, no; deer mice only; not included in patch quitting) |
|  | Deer mouse | Presence/absence of deer mice (yes, no; woodrat only; not included in patch quitting) |
| Other foragers (Stages 2-4) | Nontarget visits | Number of visits to a patch in one of four forms: A) all woodrats, B) nontarget woodrats, C) deer mice, or D) nontarget woodrats and deer mice |
|  | Nontarget foraging | Cumulative time spent in patch (s) in one of four forms: A) all woodrats, B) nontarget woodrats, C) deer mice, or D) nontarget woodrats and deer mice |

**Appendix S2 – Supplementary results**

| **Table S2.** — Results of (generalized) logistic, negative binomial, and linear mixed effects models quantifying foraging behavior for dusky-footed woodrats (*Neotoma fuscipes*) in the central Sierra Nevada, California, USA. Significant relationships are bolded at α = 0.05 and italicized at α = 0.1. | | | | | | | | | | | | | | |
| --- | --- | --- | --- | --- | --- | --- | --- | --- | --- | --- | --- | --- | --- | --- |
| Variable | Visitation | |  | # Patch visits | |  | Patch residence | |  | Vigilance | |  | GUD | |
|  | β ± SE | *p* |  | β ± SE | *p* |  | β ± SE | *p* |  | β ± SE | *p* |  | β ± SE | *p* |
| Intercept | 0.96 ± 1.06 | 0.368 |  | 1.44 ± 0.17 | **<0.001** |  | 190.59 ± 52.44 | **<0.001** |  | 3.67 ± 0.22 | **<0.001** |  | 13.44 ± 1.17 | **<0.001** |
| Forest type | 2.76 ± 1.43 | *0.054* |  | 0.35 ± 0.20 | *0.07* |  | -19.69 ± 69.61 | 0.778 |  | -1.56 ± 0.28 | **<0.001** |  | -4.42 ± 1.45 | **0.005** |
| Treatment | 1.87 ± 0.59 | **0.002** |  | 0.29 ± 0.10 | **0.002** |  | 29.86 ± 53.98 | 0.581 |  | -0.83 ± 0.25 | **0.001** |  | -3.77 ± 1.19 | **0.002** |
| Cover |  |  |  |  |  |  | 6.66 ± 33.54 | 0.844 |  | 0.18 ± 0.13 | 0.171 |  | 1.19 ± 0.85 | 0.175 |
| Understory |  |  |  |  |  |  |  |  |  |  |  |  |  |  |
| Sex | -3.11 ± 1.38 | **0.025** |  | -0.46 ± 0.23 | *0.054* |  |  |  |  |  |  |  |  |  |
| Age |  |  |  |  |  |  |  |  |  |  |  |  | 2.95 ± 1.38 | **0.047** |
| Nontarget woodrat | 1.07 ± 0.50 | **0.031** |  |  |  |  |  |  |  |  |  |  |  |  |
| Nontarget woodrat PRT |  |  |  | 0.18 ± 0.06 | **0.002** |  | 72.65 ± 20.43 | **0.001** |  |  |  |  | -1.70 ± 0.50 | **<0.001** |
| # Nontarget woodrat visits |  |  |  |  |  |  |  |  |  | 0.30 ± 0.07 | **<0.001** |  |  |  |
| Forest type * Cover |  |  |  |  |  |  | 140.29 ± 56.00 | **0.018** |  | -0.26 ± 0.20 | 0.199 |  | -5.62 ± 1.35 | **0.001** |
| Forest type * Treatment | -2.82 ± 0.94 | **0.003** |  |  |  |  | 153.29 ± 73.83 | **0.039** |  | 0.86 ± 0.33 | **0.01** |  | 3.50 ± 1.54 | **0.025** |
| Effects (β) and their standard error (SE) are displayed as well as *p* values from model comparisons. All continuous variables have been standardized, and the proportion of time vigilant was square-root transformed. The reference level for categorical modalities are mature for forest type, risky for treatment, female for sex, 'adult' for age class, and no for visitation by nontarget woodrat. Variables that are present on Table 1 but missing here were still tested; covariates that were not present in any final model sets are not included on this table for easier interpretability. | | | | | | | | | | | | | | |

| **Table S3.** — Results of (generalized) logistic, negative binomial, and linear mixed effects models quantifying foraging behavior for deer mice (*Peromyscus* spp.) in the central Sierra Nevada, CA, USA. Significant relationships are bolded at α = 0.05 and italicized at α = 0.1. | | | | | | | | | | | |
| --- | --- | --- | --- | --- | --- | --- | --- | --- | --- | --- | --- |
| Variable | Visitation | |  | # Patch visits | |  | Patch residence | |  | GUD | |
|  | β ± SE | *p* |  | β ± SE | *p* |  | β ± SE | *p* |  | β ± SE | *p* |
| Intercept | 0.08 ± 0.52 | 0.874 |  | 2.47 ± 0.16 | **<0.001** |  | 160.41 ± 64.91 | 0.018 |  | 7.91 ± 0.86 | **<0.001** |
| Forest type |  |  |  |  |  |  | 97.55 ± 99.21 | 0.333 |  |  |  |
| Treatment |  |  |  | 0.43 ± 0.14 | **0.002** |  | 388.81 ± 68.2 | **<0.001** |  | -3.64 ± 1.01 | **0.001** |
| Cover |  |  |  |  |  |  |  |  |  | -1.17 ±0.62 | *0.099* |
| Understory |  |  |  | -0.35 ± 0.14 | **0.015** |  |  |  |  |  |  |
| Forest type * Treatment |  |  |  |  |  |  | -312.5 ± 100.33 | **0.002** |  |  |  |
| Treatment * Understory |  |  |  | 0.44 ± 0.14 | **0.002** |  |  |  |  |  |  |
| Effects (β) and their standard error (SE) are displayed as well as *p* values from model comparisons. All continuous variables have been standardized. The reference level for categorical modalities are 'young' for forest type and 'safe' for treatment. Variables that are present on Table 1 but missing here were still tested; covariates that were not present in any final model sets are not included on this table for easier interpretability. | | | | | | | | | | | |


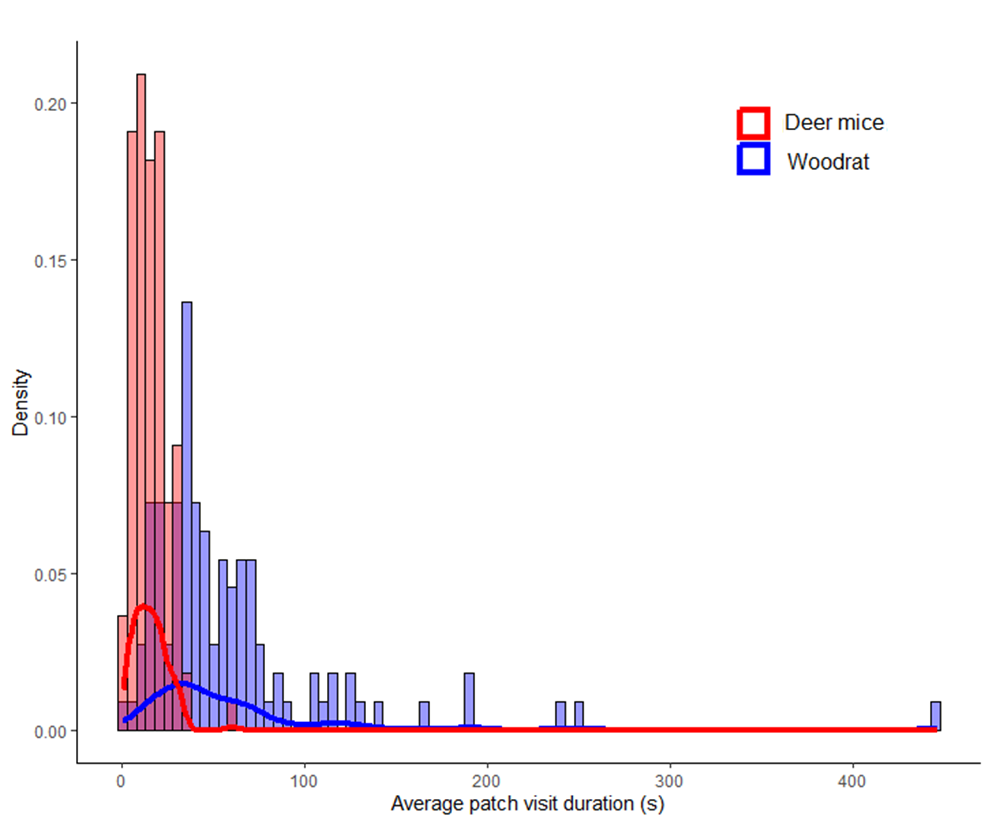


**Figure S1:** Distribution of nightly values for average patch visit duration among dusky-footed woodrats (*Neotoma fuscipes*) and deer mice (*Peromyscus* spp)*.*


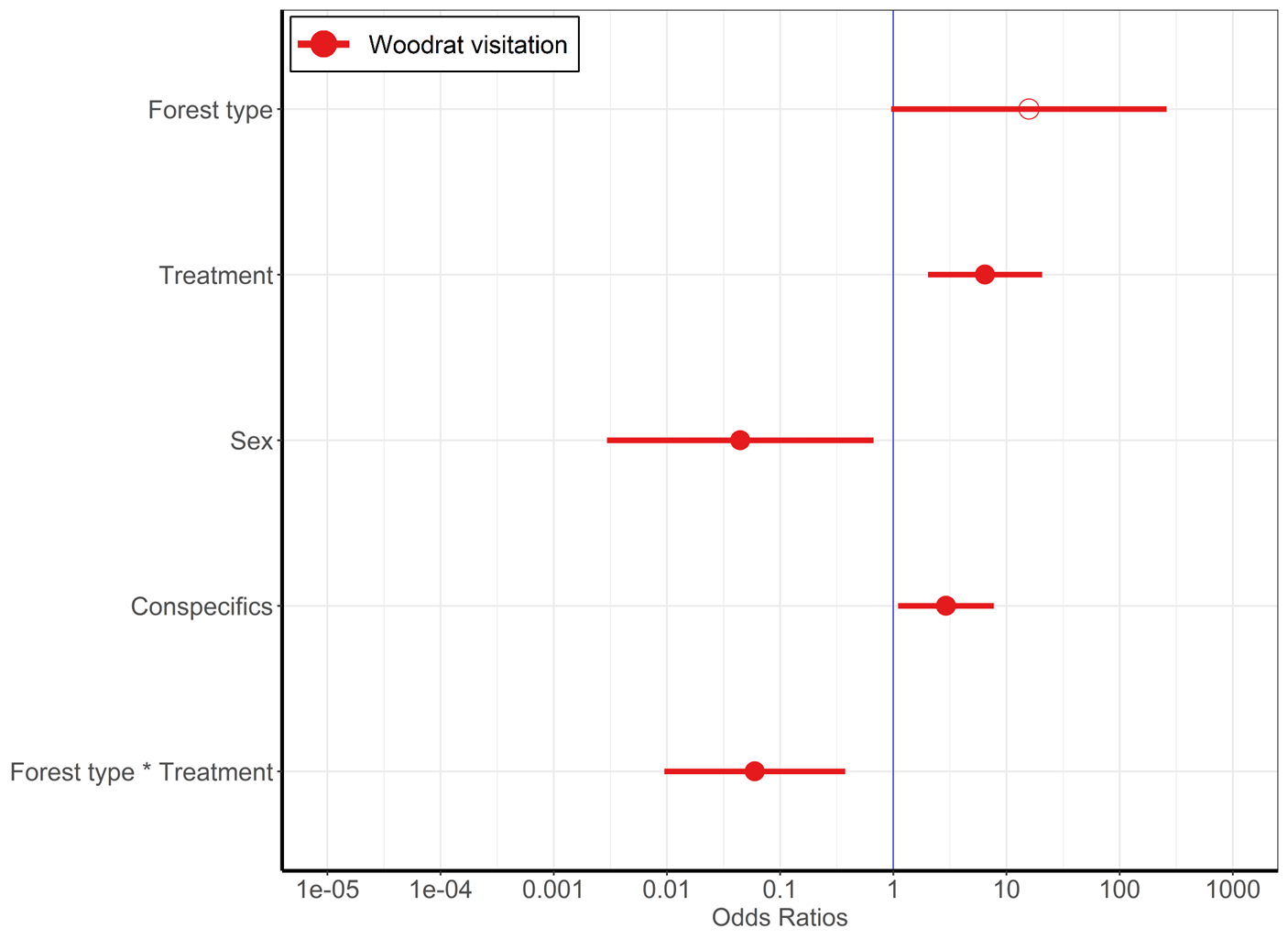


**Figure S2:** Odds ratios for patch visitation probabilities (Stage 1) among dusky-footed woodrats (*Neotoma fuscipes*) from the best-supported mixed effects logistic regression model and associated 95% confidence intervals with significance denoted by solid circles. The reference level for categorical modalities are ‘mature’ for forest type, 'risky' for treatment, 'female' for sex, and 'no' for visitation by nontarget woodrat. Odds ratios for deer mice (*Peromyscus spp.*) visitation are not displayed because the top model was the intercept model.
